# Rational Design of a Potential New Nematicide Targeting Chitin Deacetylase

**DOI:** 10.1021/acs.jafc.3c05258

**Published:** 2024-01-24

**Authors:** Maria Galvez-Llompart, Riccardo Zanni, David Vela-Corcía, Álvaro Polonio, Facundo Perez-Gimenez, Jesús Martínez-Cruz, Diego Romero, Dolores Fernández-Ortuño, Alejandro Pérez-García, Jorge Galvez

**Affiliations:** † Department of Preventive Medicine and Public Health, Food Science, Toxicology and Forensic Medicine, Faculty of Pharmacy, University of Valencia, Burjassot, Valencia 46100, Spain; ‡ Molecular Topology and Drug Design Unit. Department of Physical Chemistry, University of Valencia, Burjassot, Valencia 46100, Spain; § Department of Microbiology, Faculty of Science, Instituto de Hortofruticultura Subtropical y Mediterránea La Mayora, IHSM-UMA-CSIC, University of Málaga, Málaga 29071, Spain

**Keywords:** nematicide, chitin deacetylase, *Caenorhabditis elegans*, QSAR, molecular topology

## Abstract

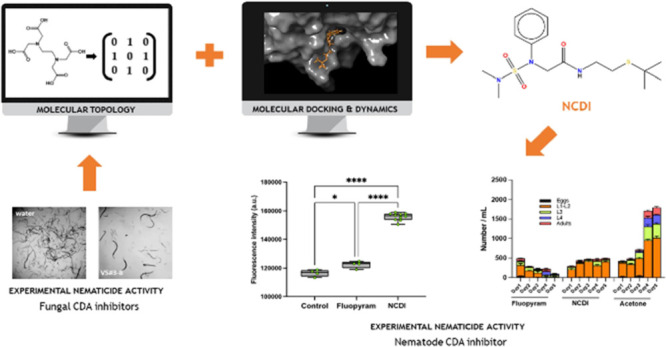

In a previously published study, the authors devised a molecular topology QSAR (quantitative structure–activity relationship) approach to detect novel fungicides acting as inhibitors of chitin deacetylase (CDA). Several of the chosen compounds exhibited noteworthy activity. Due to the close relationship between chitin-related proteins present in fungi and other chitin-containing plant-parasitic species, the authors decided to test these molecules against nematodes, based on their negative impact on agriculture. From an overall of 20 fungal CDA inhibitors, six showed to be active against *Caenorhabditis elegans*. These experimental results made it possible to develop two new molecular topology-based QSAR algorithms for the rational design of potential nematicides with CDA inhibitor activity for crop protection. Linear discriminant analysis was employed to create the two algorithms, one for identifying the chemo-mathematical pattern of commercial nematicides and the other for identifying nematicides with activity on CDA. After creating and validating the QSAR models, the authors screened several natural and synthetic compound databases, searching for alternatives to current nematicides. Finally one compound, the N2-(dimethylsulfamoyl)-*N*-{2-[(2-methyl-2-propanyl)sulfanyl]ethyl}-N2-phenylglycinamide or nematode chitin deacetylase inhibitor, was selected as the best candidate and was further investigated both in silico, through molecular docking and molecular dynamic simulations, and in vitro, through specific experimental assays. The molecule shows favorable binding behavior on the catalytic pocket of *C. elegans* CDA and the experimental assays confirm potential nematicide activity.

## 1 Introduction

Chitin is a polysaccharide that is an important structural component in the cuticles of many invertebrates, including nematodes. The cuticle is a protective outer layer that covers the nematode’s body and plays a crucial role in maintaining its shape and integrity. Chitin is a major constituent of the cuticle and provides both strength and flexibility to this layer. In addition to its structural role, chitin is also involved in various physiological functions in nematodes.^1,2^ For example, it is an important component of the eggshell, which protects the developing embryo from environmental stresses.^3,4^ Chitin is also a key component of the pharyngeal grinder, a specialized structure in the nematode digestive system that grinds food particles.^5^ Furthermore, chitin is a target for various enzymes produced by parasites and predators that feed on nematodes.^6^ Enzymes that degrade chitin, such as chitinases, can break down the nematode’s protective cuticle and compromise its integrity, making it vulnerable to attack^7^; therefore, nematodes have evolved various mechanisms to protect themselves from Chitinase attack, including producing chitinase inhibitors.^8^ Overall, chitin plays an essential role in the biology of nematodes, providing structural support and protection as well as contributing to various physiological functions. The use of chitin-related inhibitors as a basis for the design of new nematicides has been an area of active research and development in recent years. Chitin-related inhibitors are compounds that prevent the synthesis or deposition of chitin in the cuticle of nematodes, thereby disrupting their growth and development. These compounds have been explored as potential nematicides, which are chemicals used to control nematode populations that can cause significant damage to crops and other plants.^9−11^ The use of chitin synthesis inhibitors as nematicides has several advantages over those of conventional chemical nematicides. First, chitin-related inhibitors are highly specific to nematodes and have low toxicity to other organisms, including humans and wildlife, with the exception of insects. Second, they have a low environmental impact, as they degrade rapidly and do not accumulate in the soil or water.^12,13^

To design new nematicides and reduce the potential risk of resistance development, new mechanisms of action should be explored to expand the portfolio of nematicides currently available in the market. In this regard, the inhibition of chitin deacetylase (CDA) is a promising mode of action. Chitin deacetylases (CDAs) are enzymes that modify chitin by removing acetyl groups from *N*-acetyl-glucosamine units.^14^ According to the phenotypes previously observed by RNAi silencing experiments,^15^ the inhibition of CDA activity may disrupt the synthesis and function of the nematode cuticle, which plays a vital role in maintaining their shape and integrity, as well as protecting them from environmental stress and predators. The resulting damage to the cuticle can lead to dehydration, loss of structural integrity, and increased susceptibility to infections and environmental stress. In conclusion, the inhibition of CDA represents a promising mechanism of action for nematicides, offering a specific and effective approach to control nematode populations affecting, for example, vegetable crops.^16^

Several strategies can be employed in the development of synthetic chitin-related inhibitors, including the modification of natural chitin biosynthesis inhibitors, high-throughput screening (HTS), structure–activity relationship (SAR) studies, and rational design of novel inhibitors. In this last group stands quantitative structure–activity relationship (QSAR), which is a computational method used in agrochemistry to design and predict the activity of new chemical compounds. Ideally, QSAR could be used to design new compounds that effectively target the chitin modification pathway in nematodes, CDA to be precise, leading to their death.^17^ In the present work, the authors developed a molecular topology QSAR-based strategy to rationally design novel nematicides with CDA inhibitory activity.

The molecular topology approach focuses on the use of topological and topo-chemical descriptors to describe molecules, giving a chemo-mathematical interpretation of the relationship between a structure and its biological properties, by means of graph theory, which determines the connectivity of the atoms within the molecule and how it relates with different physicochemical properties. The value of this kind of descriptors is not altered by the specific molecule 3D conformation.^18−21^ To develop the QSAR strategy, the authors started from the initial data set of fungal CDA inhibitors identified in a previous work (Table S1).^17^ Starting from the hypothesis that CDA is also present in nematodes, which are chitin-containing organisms, a set of experiments on nematode *Caenorhabditis elegans* were carried out to determine the nematicide activity of the 20 compounds previously identified as fungal CDA inhibitors. From an overall of 20 compounds, six resulted to be active on nematodes. The experimental data were collected to train an algorithm center in identifying nematicides with CDA inhibitory activity. In addition, the literature was scrapped, retrieving information on commercial nematicides for training another algorithm focused on determining the chemo-mathematical pattern associated with commercial nematicides.

**Figure 1:**
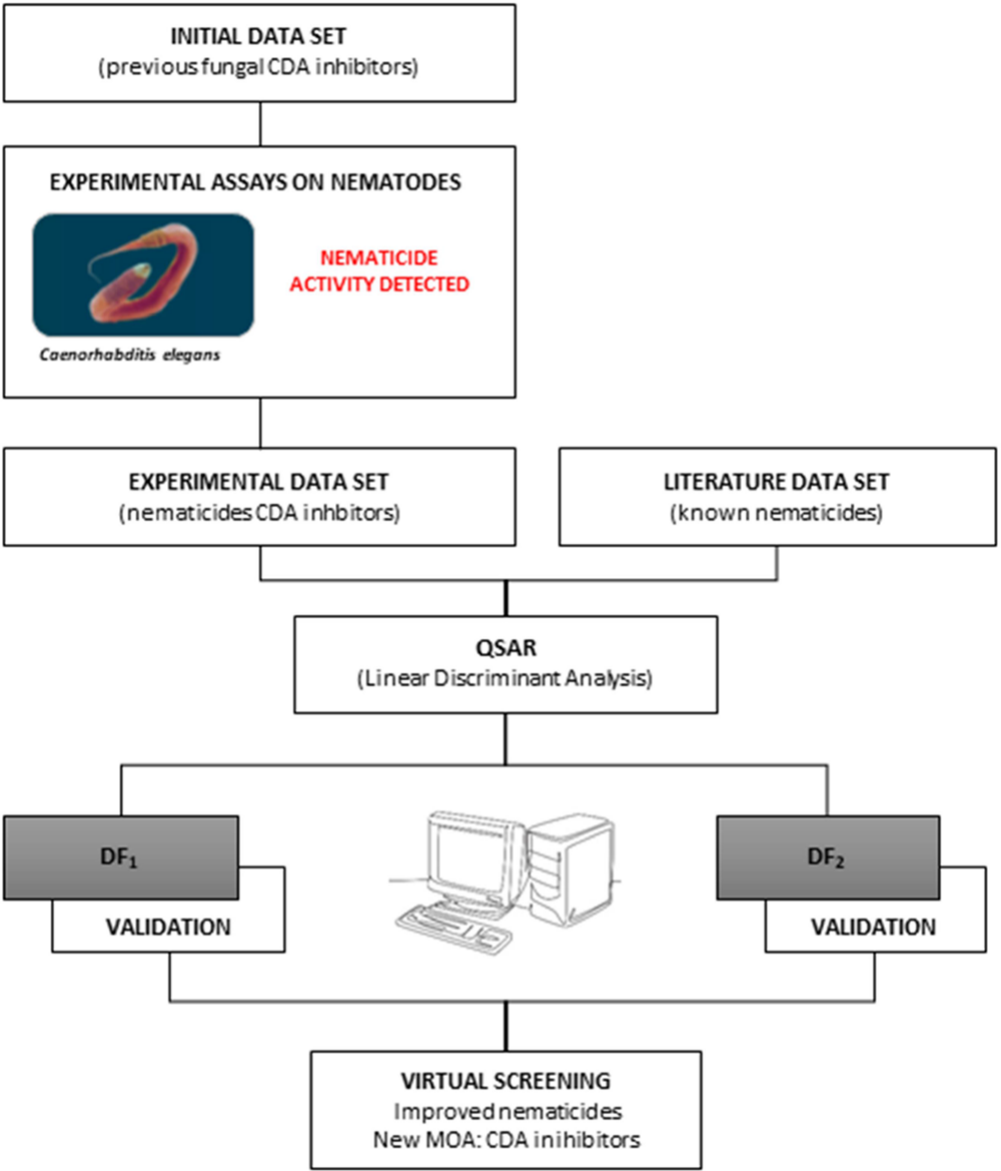
Workflow of the QSAR strategy developed for the identification of new potential nematicides with CDA inhibitory activity.

Linear discriminant analysis was employed for the development of the predictive QSAR equations. Models were validated and finally used for the virtual screening of different commercial databases, searching for new, potential nematicides, with activity against CDA enzyme. Figure 1 shows a schematic representation of the workflow followed in this study.

## 2 Experimental Methodology

### 2.1 Chemo-Mathematical Characterization of the Molecules

Graph theory was applied to calculate topological and topo-chemical descriptors, codifying information about the molecular structures in a purely numerical way. The 2D structures of the molecules used in this study were drawn using ChemDraw Ultra (version 10.0) and characterized by a set of different constitutional, topological, and topo-chemical indices (Tis). Calculation of descriptors was performed using the open-source Mordred software.^22^

### 2.2 Statistical Modeling Techniques

#### 2.2.1 Linear Discriminant Analysis (LDA)

LDA (linear discriminant analysis) is a powerful pattern recognition method used to classify molecules based on their nematicide activity. It achieves this by using a linear combination of variables, such as topological indices (TIs), to distinguish between active and inactive groups. The Mahalanobis distance is used to determine the distance of each compound from the mean of all cases in a particular group, and the Wilks parameter λ is used to assess the reliability and effectiveness of the discriminant function. The Fischer–Snedecor parameter (*F*) is employed to select the most informative variables using a stepwise strategy. These calculations were performed using STATISTICA 9.^23^

### 2.3 Nematicidal Distribution Diagrams (NDD)

To aid in the interpretation of the results, nematicidal activity distribution diagrams (NDD) were created. These diagrams plot the expectancy of activity versus the numerical outputs of the discriminant function (DF) for a particular biological activity.^24^ Expectancy of activity (Ea) is defined as *a*/(*i* + 1), where *a* and *i* are the number of active and inactive compounds in a particular interval of DF values. Similarly, the expectancy of inactivity (Ei) is defined as *i*/(*a* + 1). These diagrams help to identify the DF intervals, where there is a maximum probability of activity or inactivity. The LDA analysis generated two discriminant functions, DF_1_ and DF_2_, which were used to classify the compounds based on their nematicide activity. Overall, LDA is a powerful tool for classifying molecules based on their biological activity and can be used to streamline the drug discovery process.

### 2.4 Topological Models’ Validation

Since the initial data set for the first discriminant model (DF_1_) was very small with *n* = 20 (eq 1), internal validation or cross-validation with a leave-one-out procedure (LOO) was used to test the model’s robustness and reliability.^25^ In the LOO algorithm, one case is eliminated from the data set, and then the discrimination analysis, with the N-1 remaining cases and the original descriptors (the ones selected in the model), is performed again. The corresponding classification label value for the removed case is then predicted. For DF_2_, randomly 25% of the training set was employed as an external test set.

### 2.5 Virtual Screening

To discover a novel type of nematicides that exhibits activity against CDA, we screened various databases containing both natural and synthetic molecules, such as Lotus,^26^ Sigma-Aldrich, and eMolecules. Additionally, we used the open-source web ADMELAB2.0^27^ to calculate the ROA (acute oral toxicity in rats) value to assess the potential toxicity of new nematicide candidates.

### 2.6 Molecular Docking

Due to the unavailability of *C. elegans* CDA, AlphaFold server was employed to construct the 3D model of the mature CDA protein of *C. elegans* (H2L042).^28,29^ Once the model was completed, potential binding sites for small-molecule ligands are automatically searched on the whole CDA protein employing the SiteMap tool from Schrodinger. The program maps and scores regions on the protein surface that are likely to accommodate a ligand. The top-ranked binding site by SiteMap was employed for the molecular docking simulations on the CDA protein of *C. elegans* (H2L042). Then, the grid was generated using the Receptor Grid Generation tool in the Grid-based Ligand Docking with Energetics (Glide) module of Schrödinger suite (version 2022–4). Before docking simulations, both ligand and protein were prepared keeping the settings at default, by using LigPrep and Protein Preparation Wizard from the Maestro, respectively. Docking calculations were performed using the standard precision (SP) scoring function (Glide SP) of the Schrödinger software suite molecular modeling package (version 2022–4), using default parameters unless otherwise reported.

### 2.7 Molecular Dynamics

MD simulations were performed using the Desmond module of the Schrodinger suite (version 2022–4). All systems were solvated in an orthorhombic box (a margin of 10 Å between the solute and the side of the box was used in each dimension) with explicit TIP3P water molecules. All systems were neutralized, and an ionic salt concentration of 0.15 M of Na^+^ and Cl^–^ was added. Atomistic interactions were calculated with the OPLS4 force field (Schrödinger 2022–4). After the construction of the solvent environment, each complex system was composed of about 78437 atoms. Before equilibration and the long-production MD simulations, the systems were minimized and pre-equilibrated using the default relaxation routine implemented in Desmond. A multiple time-stepping of 2, 2, and 6 fs was used. The system equilibration was done via NVT and NPT ensembles using the SHAKE algorithm and by bringing the temperature to 300 K and pressure to 1 bar. Then, the systems were submitted in 10 and 50 ns MD simulations for equilibration and production MD runs for each system. Finally, a 50 ns nonconstrained MD simulation was performed for the system, and the coordinates were saved for every 5 ps.

### 2.8 Reactive Oxygen Species Assay

Reactive oxygen species (ROS) production triggered by 1 mM NCDI was analyzed. As positive and negative controls, 0.6 mM Floupyram and solvent 1.5% acetone were used, respectively. Reactive oxygen species detection was performed by means of dihydrorhodamine 123 (DHR123, Sigma-Aldrich). DHR123 was added in a proportion of 1:1000 to a *C. elegans* N2 suspension in M9 buffer, followed by 15 min incubation at room temperature in the dark. ROS production was measured by taking records every 30 min at 25 °C for 24 h. All measurements were performed in a microplate reader, FLUOstar Omega (BMG LABTECH), using the 488 filter.

### 2.9 *Caenorhabditis elegans* Toxicity Assay

To investigate the toxicity against nematodes, a *C. elegans* plate-based toxicity assay was used.^15^*C. elegans* were cultivated on 10 mm Petri dishes with NGM agar^30^ seeded with 200 μL of *Escherichia coli* OP50 from an overnight culture and maintained at 20 °C. To recover the nematode populations, plates were washed twice with 600 μL of M9 buffer and filtered through Miracloth filters.^30,31^ Each well of a 24-well plate containing 500 μL of NGM agar was seeded with 20 μL of *Escherichia coli* OP50 from an overnight culture. The bacterial lawn was allowed to grow at room temperature for 1 day.^30^ To conduct the toxicity assay, in each well plate, a 75 μL drop of the different compound solutions was added, and the mixture was dried on the agar at room temperature for 30 min. A solution of 1.5% acetone was used as a negative control. Then, each well was inoculated with 40 μL of the nematode populations filtered above. After 5 days of incubation of the well plates at 20 °C, each well was washed with 100 μL of M9 buffer. Subsequently, ten drops of 3 μL were used to count the number of eggs and the number of nematodes in different stages of development (L1, L2-L3, and L4) under a microscope to evaluate the effect of compounds.

## 3 Results and Discussion

### 3.1 Nematicidal Effect of Fungal Chitin Deacetylase Inhibitors

**Figure 2:**
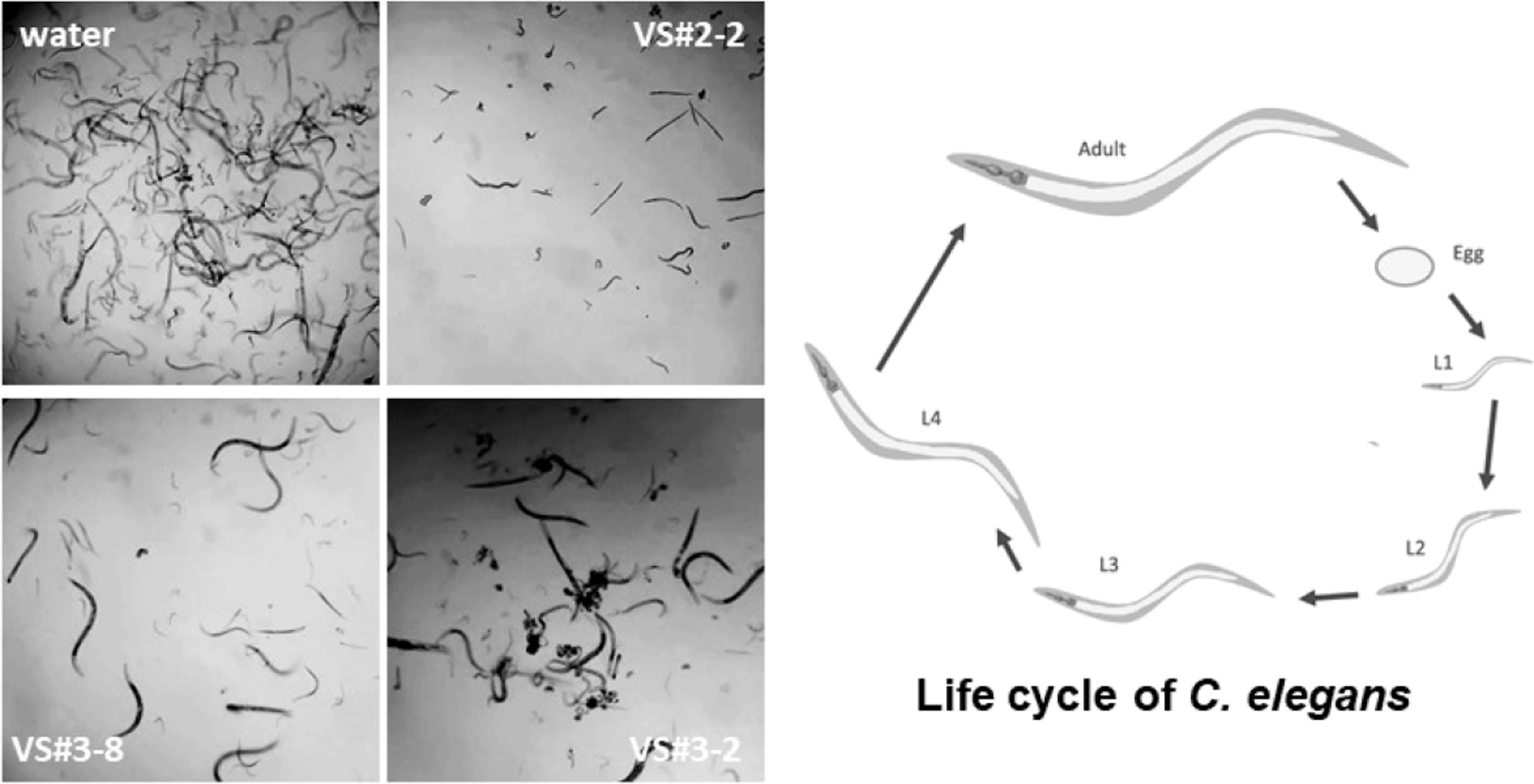
Nematicidal effect of the three most active compounds against *C. elegans*. The life cycle of *C. elegans* is also included. See Table S1 for details.

As indicated above, CDA inhibitors previously identified for their abilities to suppress fungal diseases and obtained by a molecular topology approach^17^ were tested against the model nematode *C. elegans* to explore the possibility that CDA inhibitors may also target nematode CDA. Table S1 shows the nematicidal effect on *C. elegans* of the fungal chitin deacetylase inhibitors identified by molecular topology. All of the compounds tested showed some level of activity on some of the larval stages evaluated, identifying several compounds that showed a strong effect on the development of *C. elegans*. This result is consistent with the phenotype of delay in developmental timing associated with the RNAi silencing of CDA-coding genes.^15^ The compounds with the strongest nematicidal activity according to the statistical analysis performed were VS#**2**-2, VS#**3**-1, VS#**3**-2, VS#**3**-4, VS#**3**-6, and VS#**3**-8. Figure 2 shows representative pictures of the nematicidal effects of the three top compounds tested against *C. elegans*. Compared to the negative control (water), these compounds provoked a clear reduction in the development of nematodes.

### 3.2 Development and Validation of the QSAR Classification Models

Once the nematicide activity for the potential CDA inhibitors was proved, the experimental data was collected and used to develop a molecular topology QSAR algorithm (DF_1_), for the identification of new, improved nematicides, following the CDA inhibition MOA. Additionally, data from the literature were scrapped, to collect a database of known nematicides, in order to design a model for the determination of the nematicides chemo mathematical pattern (DF_2_). By combining both algorithms, potential nematicides with CDA activity are expected to be identified through virtual screening methods.

The first discrimination function, the one based on experimental results, DF_1_, is reported:
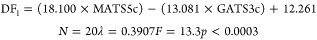
1

According to this equation, a compound will be classified as active (potential nematicide with CDA inhibitory activity) if DF_1_ > 0 and as inactive if DF_1_ < 0. Table S2 shows the DF_1_ values for each training set compound. As can be seen in Table 1, the model shows good sensitivity, with 83% correct classification for the active set, and strong specificity, with 93% correct classification for the inactive set, for an overall correct classification of 90%. This is desirable because high levels of specificity mean lower probabilities of false activity when performing virtual screening strategies. In other words, when selecting new potential nematicides, some active compounds may be lost during the screening, but activity will be assured for the final selection.

**Table 1:** Classification Matrix for the DF_1_ Training Set and DF_1_ Internal Validation (LOO)^a^

**training set**	**correct classification (%)**	**compound class. as active**	**compound class. as inactive**
active	83	5	1
inactive	93	1	13
LOO set			
active	83	5	1
inactive	93	1	13

a Class.: classification.

In eq 1, MATS5c represents the Moran coefficient of lag 5 weighted by Gasteiger charge, and GATS3c represents the Geary coefficient of lag 3 weighted by Gasteiger charge.

**Figure 3:**
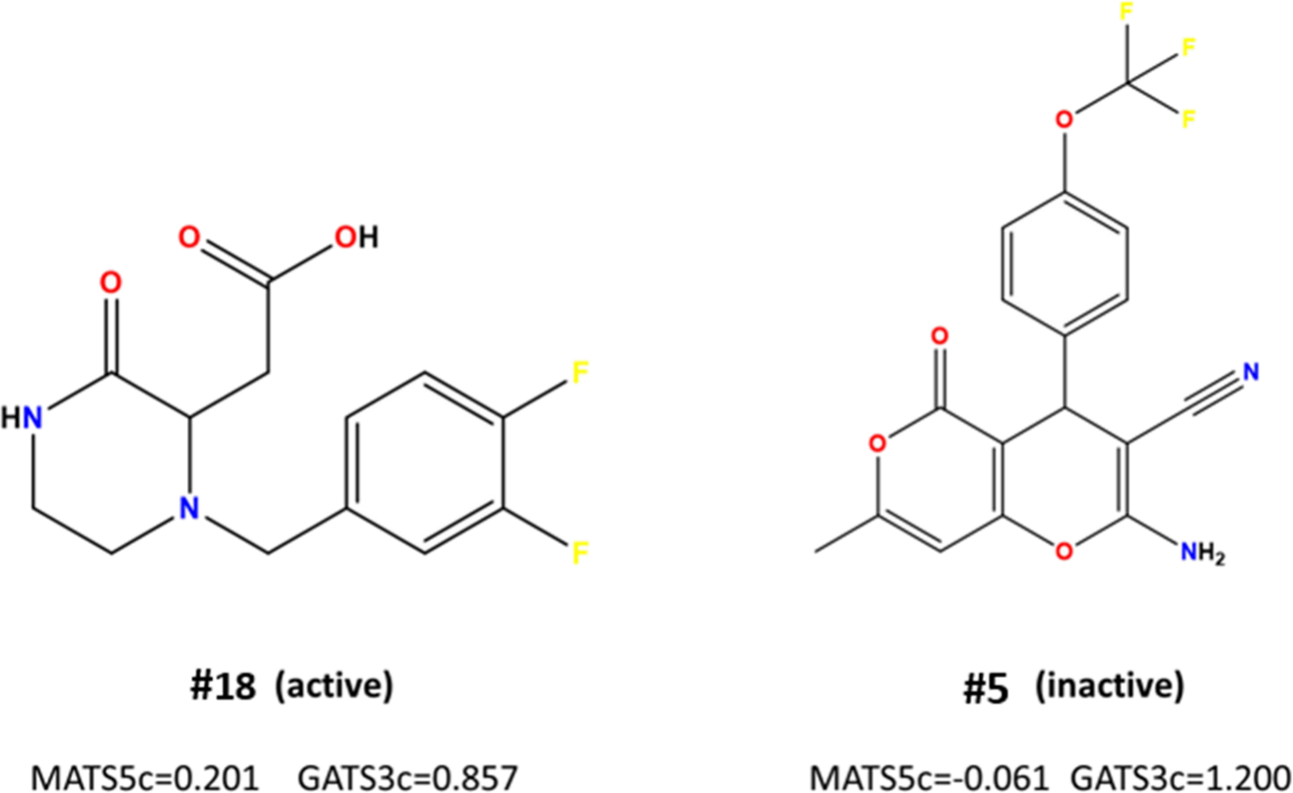
Example of topological active and inactive chemical structures for the training set, with respective MATS5c and GATS3c values.

The values of the topological descriptors for each compound of the training set are reported in Table S2. MATS5c descriptor is a Moran autocorrelation index, considering the Gasteiger charge of atoms at a topological distance of 5. As shown in Figure 3, molecules with higher electronegative (EN) atoms connected at a topological distance of 5, as well as a greater presence of sp2 and sp3 bonds at the same distance from an atom with higher EN, will have a lower value of this descriptor. Therefore, the presence of an orbital with higher EN at a topological distance of 5 is associated with a lower value of this descriptor, as seen in #05. Similarly, according to the analysis of GATS3c, the Geary coefficient of lag 3 weighted by Gasteiger charge values indicates that the presence of atoms with higher EN at a topological distance of 3, as well as a greater presence of sp2 and sp3 type bonds at the same distance with atoms having higher EN, will contribute to adopting lower values of this index, as seen in #18.

**Figure 4:**
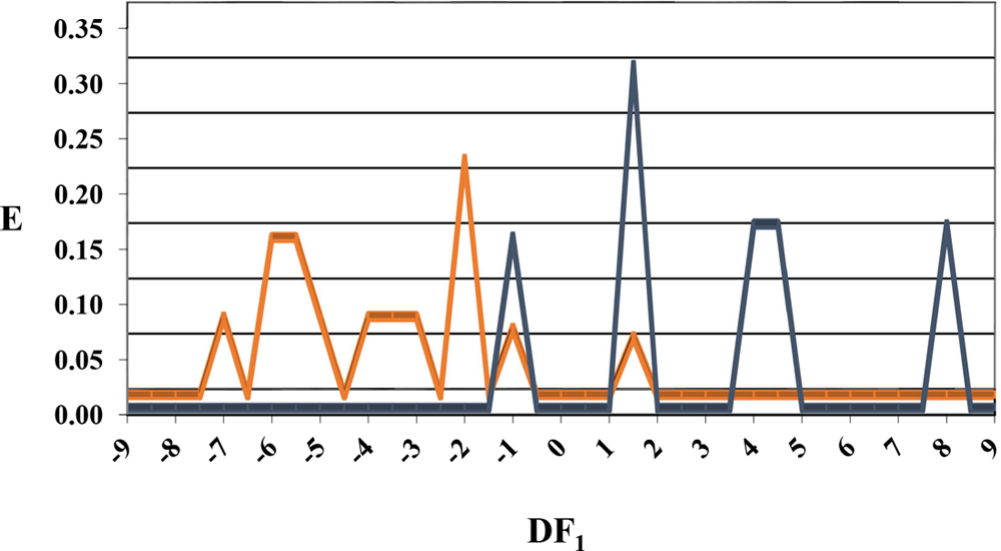
Discriminant function 1 (DF_1_) nematicidal distribution diagram (NDD). Blue peaks represent the nematicides’ CDA inhibitors distribution, while orange peaks represent the inactive.

In Figure 4, the NDD (nematicidal distribution diagram) shows that compounds with potential CDA inhibitory activity have a clear distribution in the range from 0 to +9, according to DF_1_. Hence, in this range, compounds will be classified as potential nematicides with CDA inhibitory activity.

The DF_1_ function was internally validated using a “leave one out” procedure as the low number of training set compounds (*n* = 20) is unsuitable for performing an external validation of the model (see Table S3). As can also be seen in Table 1, results in terms of classification obtained after internal validation were comparable to those of the selected model (see values of DF_1_ and probability of activity). Therefore, the model demonstrates its robustness, and its predictions do not depend on the presence of any single compound in the training set.

The second discriminant equation (DF_2_), focuses on the determination of the chemo-mathematical pattern of known and commercial nematicides. In this case, the inactive group of compounds was conformed of compounds sharing significant structural similarity with known nematicide but without expected nematicide activity (decoys).
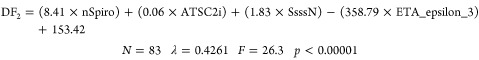
2

In eq 2, nSpiro is the number of spiro atoms, ATSCi is the centered Moreau–Broto autocorrelation index of lag 2 weighted by ionization potential, SsssN is the sum of sssN, and ETA_epsilon_3 ETA is the epsilon (type: 3) index, where ETA is the extended topochemical atom (ETA) descriptor.

According to eq 2, a compound will be classified as active (potential nematicide) if DF_2_ > 0 and as inactive if DF_2_ < 0. Table S4 shows the value of DF_2_ for each compound of the training set, as well as the value of the topological descriptors.

DF_2_ reported good sensitivity (85%) and specificity (89%), with an overall correct classification rate of 87%, as it can be seen in Table 2. Once again, the algorithm is more specific than sensitive in identifying nematocides.

**Table 2:** Classification Matrix for DF_2_ Training and External Validation Set^a^

**training set**	**correct classification (%)**	**compound class. as active**	**compound class. as inactive**
active	85	23	4
inactive	89	6	50
test set			
active	75	6	2
inactive	86	3	18

a Class.: classification.

In this particular case, an external validation test was conducted due to the availability of a larger data set (see Table S4). To achieve this, 25% of the data were excluded from the training set and used as an external test set. Once the discriminant model was developed, the test set was utilized to evaluate the accuracy of the algorithm in classifying the compounds (nematicide and decoys) never seen by the model. The results of the validation, presented in Table 2, provide valuable insights into the reliability of the predictive model, with a correct classification rate of 75% for the active compounds and 86% for the inactive ones, resulting in a model highly specific.

**Figure 5:**
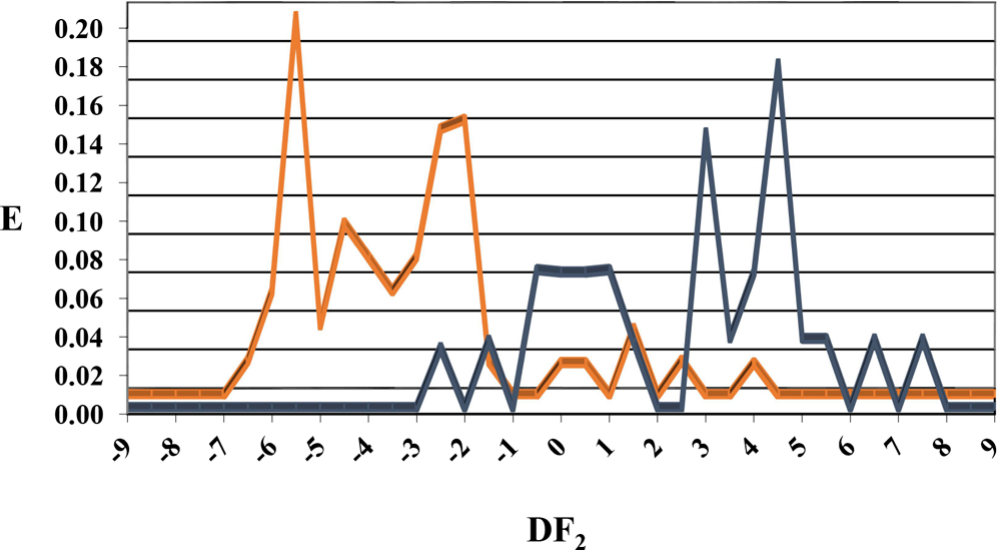
Discriminant function 2 (DF_2_) nematicidal distribution diagram (NDD). Blue peaks represent the nematicides’ CDA inhibitor distribution, while orange peaks are the inactive.

Once our model is trained and validated, a visual representation of the DF values adopted by known and commercial nematicides and decoys provides highly valuable information for future model applications. In Figure 5, is reported the nematicide distribution diagram obtained from DF_2_, where it is possible to observe how nematicide activity can be found in the range between −1 and 8, with a clear and marked distribution between 2 and 8.

After training the classification algorithm, it becomes capable of identifying compounds with nematicidal activity without the need to analyze the specific mechanism through which they exert this activity. Moreover, by selecting the most relevant molecular descriptors, the algorithm identifies the chemo-mathematical pattern of these nematicides. As a result, we will proceed to analyze the information provided by the molecular descriptors used in the DF_2_ model.

**Figure 6:**
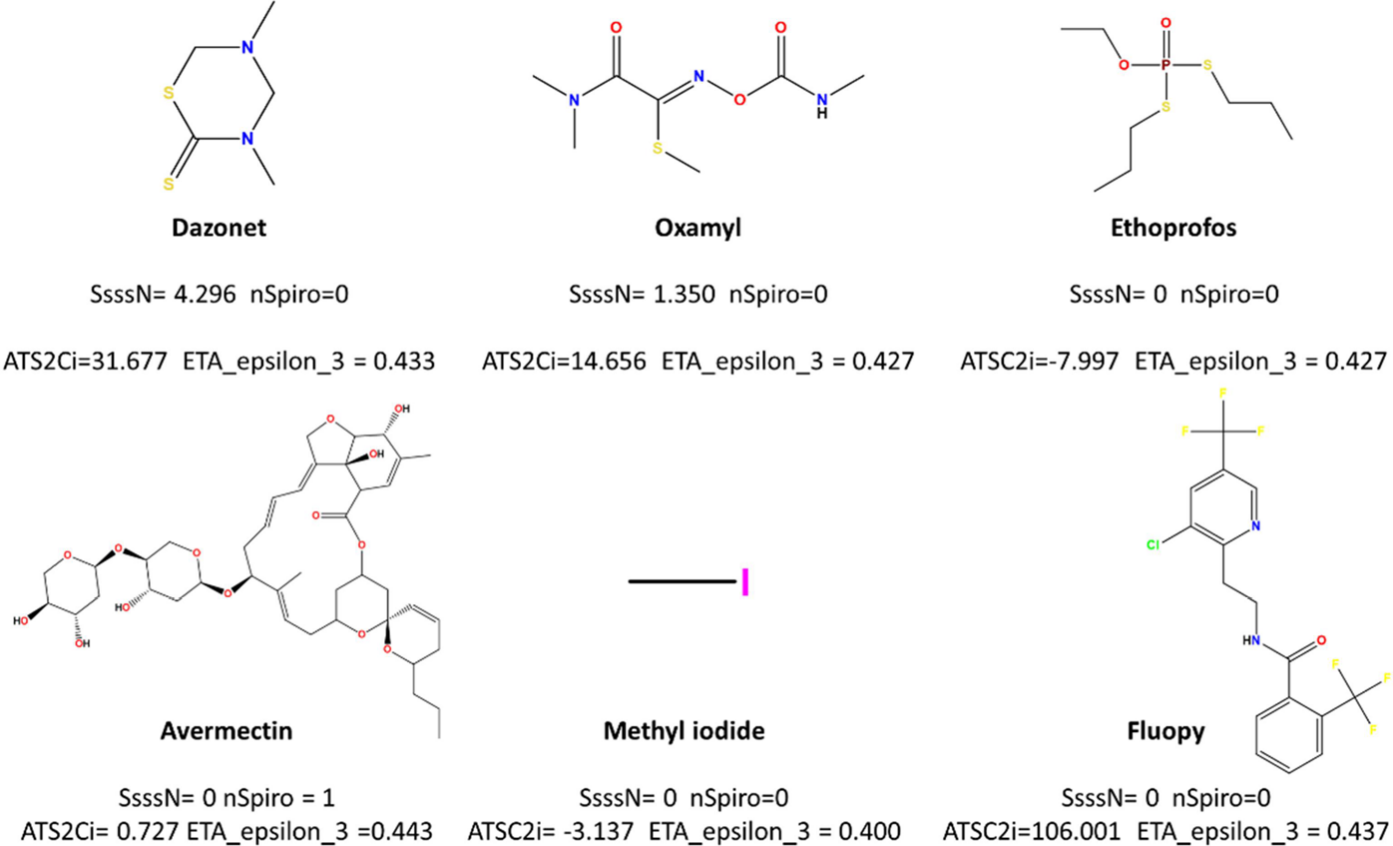
Example of commercial nematicides with their respective topological and topo-chemical descriptor values.

The descriptor nSpiro considers the presence of spiro compounds in a molecule and suggests a correlation between this group and the probability of developing nematicidal activity. However, it is not the only necessary condition to exhibit nematicide activity since only 4 out of 27 nematicides have this feature in their chemical structure. SsssN is an atom-type E-state descriptor that represents the sum of the atom-level E-state values for all of the >N– (tertiary nitrogens) group nitrogen atoms in the molecule. This descriptor increases its value as the presence of tertiary nitrogen in the molecule increases, particularly if it is within a cycle. The equation yields a positive value, indicating that the greater the number of tertiary nitrogen atoms present, the higher the expected nematicidal activity. This is one of several factors that the topological algorithm considers to be key in developing such activity. Specifically, 8 out of 27 nematicides have a group of tertiary amines in their structure, representing a desirable feature. In total, 12 out of 27 nematicides have either a spiro group or a tertiary amine in their structure, accounting for 44% of commercial nematicides. In contrast, only 5 out of 56 inactive structures (9%) follow this chemical pattern. Therefore, the presence of a spiro group or tertiary amine appears to be strongly associated with nematicidal activity, although not all nematicides possess this feature. This pattern is exemplified in Figure 6, where Dazomet, Oxamyl, and Avermectin present a spiro group or tertiary amine in their chemical structure.

The descriptor ATSC2i is the centered Moreau–Broto autocorrelation index of lag 2 weighted by the ionization potential. Compounds with atoms possessing greater ionization potential at a topological distance of 2 (e.g., Fluopyram) show higher values for this descriptor (see Table S4). As this descriptor has a direct relationship with nematicide activity, as suggested by the ATSC2i index, the presence of atoms with a higher potential for ionization at a distance of 2 seems to contribute to their nematicide activity.

The final descriptor, ETA_Epsilon_3, belongs to the category of extended topochemical indices and serves as a measure of the electronegative atom count. This descriptor considers the presence of EN atoms in the molecule, weighted by its molecular size. This could be exemplified by the azadirachtin compound, which is larger and has more EN atoms than methyl iodide and yields a higher value for this descriptor (see Table S4). Given the descriptor’s indirect relationship with nematicide activity, the trend indicates that active nematicides tend to have lower values for this descriptor (average ETA_Epsilon_3 value for training set nematicides is 0.429, whereas decoys exhibit an average value of 0.437), which is entirely reasonable.

The chemo-mathematical pattern depicted by both DF 1 and 2 will be translated into a virtual screening of chemical databases in order to identify a novel potential nematicide with CDA inhibitory activity.

### 3.3 Virtual Screening Strategy

Three databases comprising natural (Lotus) and synthetic (Sigma-Aldrich and eMolecules) compounds were screened. Different descriptors were calculated with open-source Mordred software, allowing the first filtering of molecules with potential nematicide activity through the inhibition of the CDA enzyme. After that, the value of ROA (rat oral acute toxicity) was calculated using the open-source web program ADMELAB2.0.

The overall established criteria for the selection of potential nematicides with an effect against CDA were (a) meeting the activity criteria established by NDD of DF_1_, (b) meeting the activity criteria established by NDD of DF_2_, (c) having an ROA value (<5%), (d) being commercially available, and (e) not having been previously described as nematicides in the literature. ROA was calculated because nematicides are highly toxic compounds with very low LD_50_ values. The cutoff value of ROA toxicity was established by comparing the known toxicity of commercial nematicides with the ADMELAB2.0 predictions. To give some examples, according to the software, Fluopyram, which is not toxic, has an ROA below 5%, while toxic compounds such as Aldicarb and Temik show higher ROA values above 5%. Values below 5% have been considered optimal when selecting potential nontoxic nematicides. According to all requirements, compound N2-(dimethylsulfamoyl)-*N*-{2-[(2-methyl-2-propanyl)sulfanyl]ethyl}-N2-phenylglycinamide, which will be addressed in this study as NCDI (nematode chitin deacetylase inhibitor), was selected as the most promising candidate.

In Table 3, the discriminant function values with the classification and the probability of classification of both models, along with the ROA value for NCDI, are reported. As can be seen, the potential nematicide proposed with a novel MOA (CDA inhibitory activity) has a probability of being active higher than or equal to 90% according to our model predictions (Table 3).

**Table 3:** Discriminant Function Values along with the Classification and Probability of Classification by the Models and the Predicted Rat Oral Acute Toxicity (ROA) Value for NCDI

**compound**	**DF**_**1**_	**class.**	**prob. class.**	**DF**_**2**_	**class.**	**prob. class.**	**ROA**
NCDI	2.164	A	90%	4.697	A	99%	3.5%

**Figure 7:**
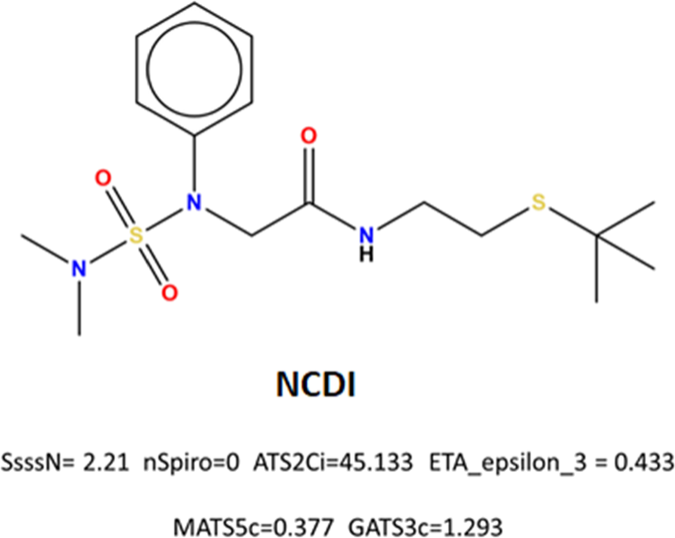
Chemical structure and topo-chemical descriptors of the potential nematicide with CDA inhibitory activity selected by the QSAR strategy.

In Figure 7, NCDI can be observed to have tertiary amines in its structure, as well as atoms at topologic distance 2 with high ionization potential, fulfilling the requirements of two key chemo mathematical patterns directly related to the nematicide activity as algorithms suggested.

### 3.4 Molecular Docking Simulation

**Figure 8:**
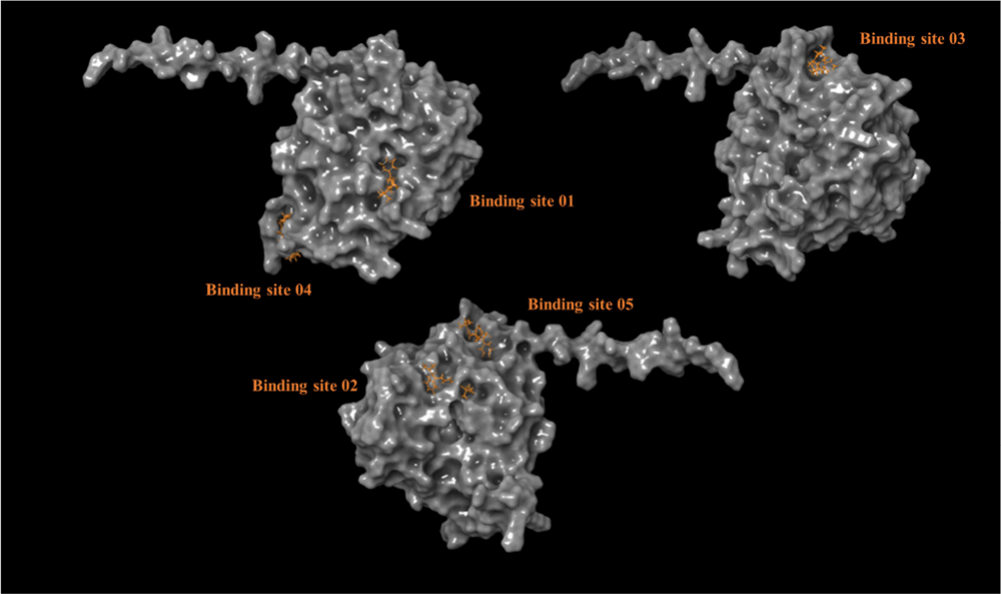
Proposed binding sites for CDA of *C. elegans* using the SiteMap tool from Schrödinger.

Because of the unavailability of the *C. elegans* CDA 3D structure, a mature protein was constructed using the AlphaFold server. Once the predicted 3D structure of *C. elegans* CDA was obtained, molecular docking simulations were carried out to predict the binding interactions of our ligand (potential nematicide with CDA inhibitory activity) with the active site of *C. elegans* CDA to confirm the mechanism of action. In Figure 8, the different potential binding sites found by SiteMap, when scanning the *C. elegans* CDA protein, are shown.

**Figure 9:**
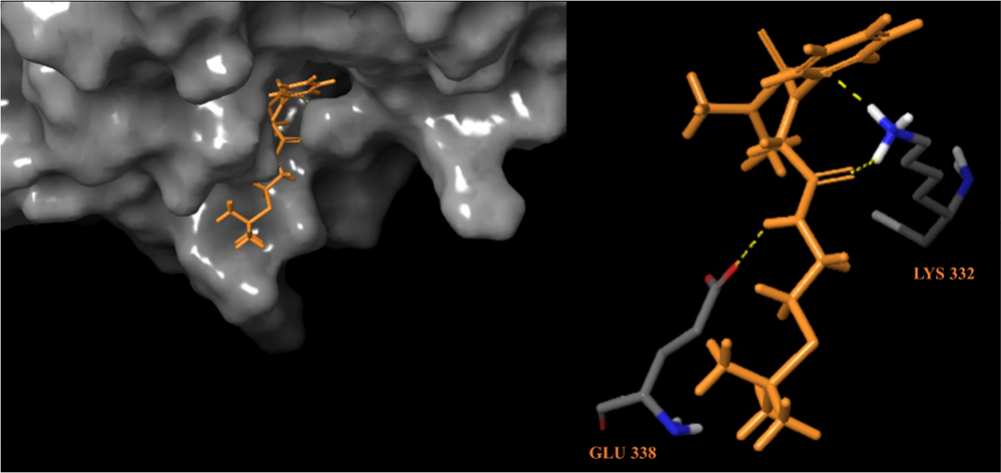
Left image shows the top-ranked binding mode of NCDI in *C. elegans*, while the right image depicts the detailed 3D interactions between the docked ligand and the protein.

Table 4 presents the docking scores of potential nematicides on five different binding sites. Notably, the results reveal that binding site number 1 has the most favorable docking score for NCDI (−3.981 kcal/mol). Therefore, we focus our analysis on the interactions between the protein and ligand at this binding site (Figure 9). Based on the molecular docking study, we suggest that our nematicide candidate inhibits the CDA enzyme by interacting with the amino acids Glu 338 (1 HB) and Lys 332 (2 HB) through the formation of hydrogen bond (HB) interactions (Figure 9). The bond formation involves hydroxy groups and nitrogen atoms.

**Table 4:** Docking Score for NCDI and CDA of *C. elegans* as the Target Protein

**binding site**	**docking score**(kcal/mol)
1	–3.981
2	–1.775
3	–3.684
4	–3.654
5	–2.433

### 3.5 Molecular Dynamics Simulations

To enhance the accuracy of the protein–ligand binding mode obtained from the docking procedure, molecular dynamics (MD) simulations were performed. By monitoring the stability of the protein–ligand complex over time, unreliable docking results can be identified and corrected.^32,33^ Although the RMSD of the protein may exhibit fluctuations during the simulation (as depicted in Figure S1), it ultimately stabilizes at a fixed value of 8–9 Å, which occurs around the 25 ns mark. Thus, to ensure the reliability of our findings, we have restricted our analysis to the time range of 25–45 ns.

**Figure 10:**
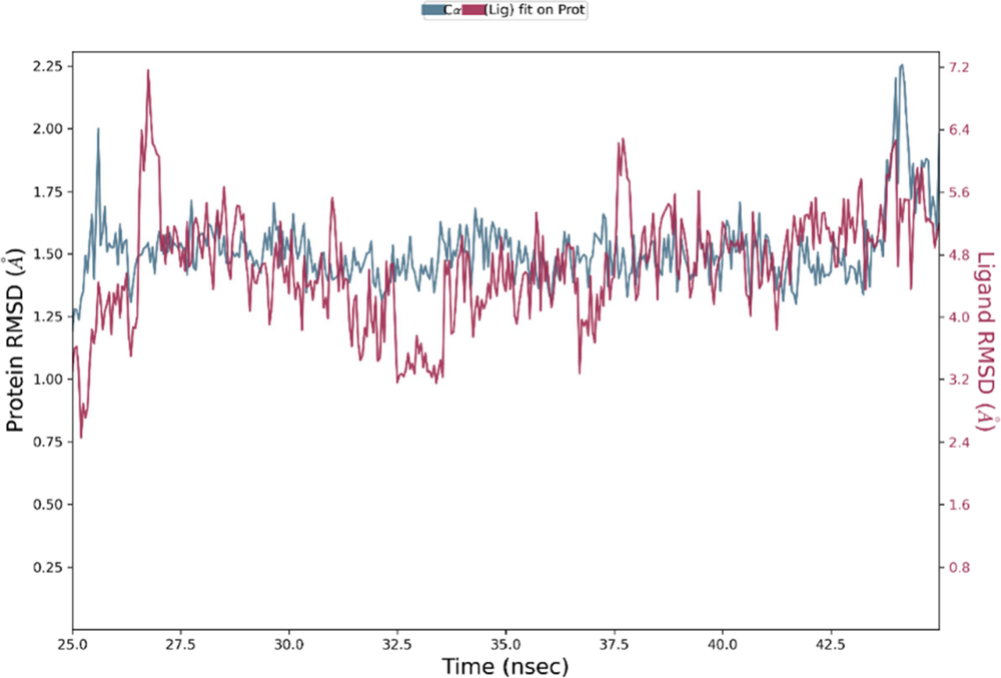
RMSD values of the Cα atoms of the CDA *C. elegans* in complex with NCDI, computed from the trajectory range 25–45 ns during MD simulations (represented by the blue lines). The RMSD values of the ligand’s heavy atoms, after being superimposed to the Cα atoms of the protein through least-squares-fit, are also depicted in purple.

**Figure 11:**
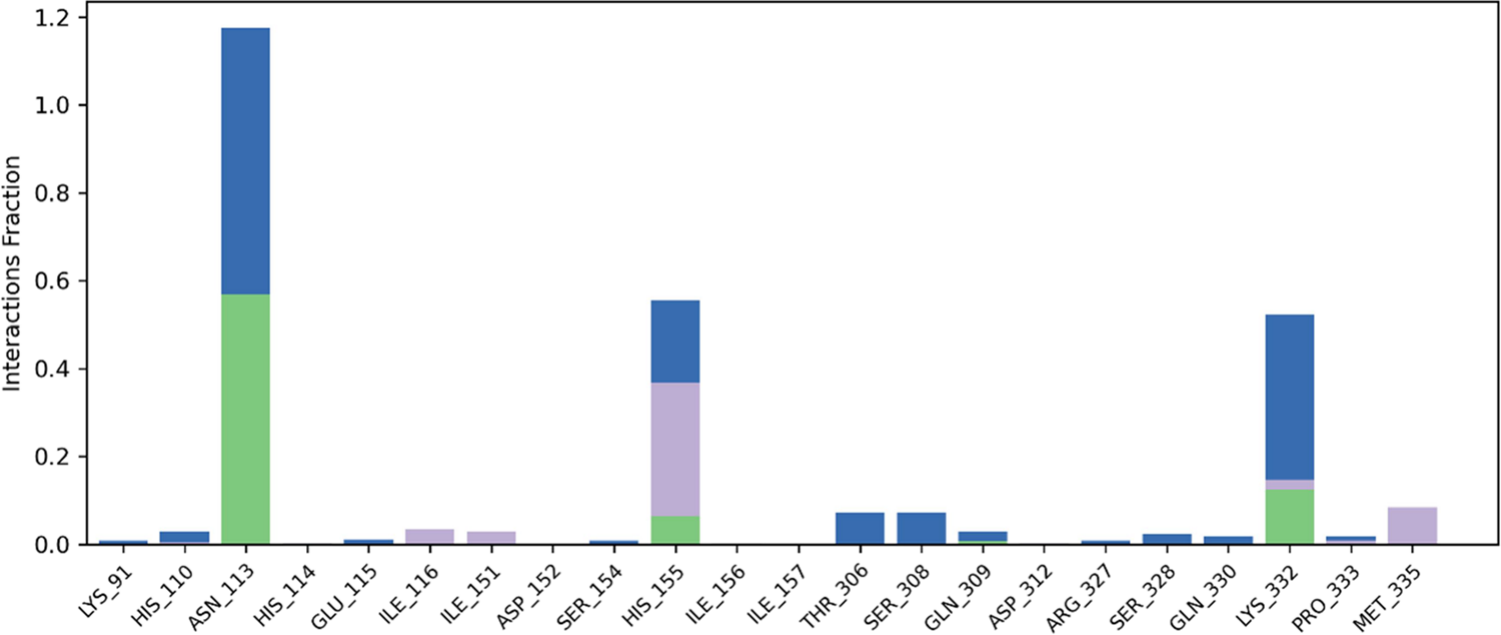
Protein–ligand interactions monitored throughout the MD simulation (simulation period of 25–45 ns). Hydrogen bonds are shown in green, water-mediated hydrogen bonds in blue and hydrophobic interactions in purple.

**Figure 12:**
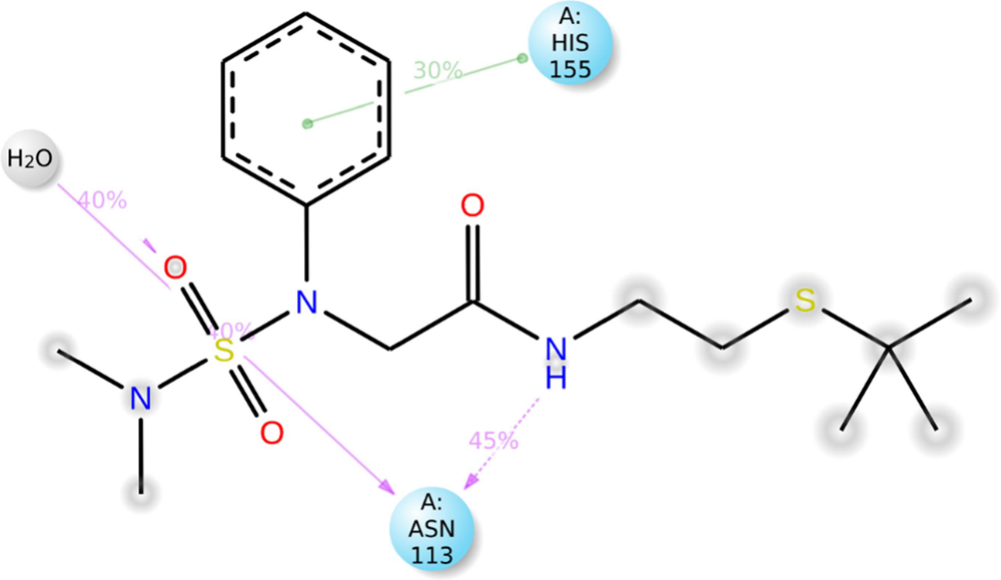
Specific atom-level interactions between NCDI and the residues of CDA *C. elegans*. Only interactions that occur for more than 30.0% of the simulation time within the selected trajectory range (25.0–45.0 ns) are depicted.

Figure 10 illustrates that during the simulation period when the CDA structure remains stable, there are only slight fluctuations in the position of the ligand within the binding pocket. By examining the per-residue protein–ligand interactions in this time range, we can conclude that NCDI maintains the key hydrogen bond interaction with Lys332 that was observed in the molecular docking analysis. Moreover, our analysis revealed additional interactions that were not detected during the docking procedure, specifically with ASN113 and HIS155 (as shown in Figures 11 and 12). Notably, ASN113 has emerged as a crucial interaction with the ligand, persisting throughout most of the simulation period. The nature of this interaction is a hydrogen bond, which appears to be critical for stabilizing the protein–ligand complex, as it is the most consistent interaction observed over time (i.e., it is the interaction that persists for the longest time during the simulation period). Specifically, Figure 12 indicates that this interaction is maintained for 40% of the simulation time. Additionally, HIS115 and NCDI engage in hydrophobic interactions (pi-pi) and a hydrogen bond for at least 30% of the analyzed simulation period.

Despite scoring functions generally not being sensitive enough to accurately describe selectivity, there is an encouraging agreement between the predictions of the QSAR models and the indications provided by the docking score and dynamics simulations. Specifically, both ligand- and structure-based approaches suggest that the selected molecule has the potential to inhibit CDA. While this information was obtained through several approximations (including a hypothetical model of the CDA nematode structure), it provides some validation of the potential mechanism of action (MOA) of our nematicide candidate. To obtain more reliable data on the MOA of this compound, it will be necessary to have access to the crystal structure of the *C. elegans* CDA protein. Nonetheless, this information was instrumental in our decision to proceed with *in vitro* biological assays to confirm the potential nematicidal activity of the NCDI compound.

### 3.6 Reactive Oxygen Species production in *C. elegans*

When nematodes are exposed to nematicides, it can lead to an increase in the level of ROS production within their cells. Elevated levels of ROS can cause oxidative stress, damaging cellular structures such as DNA, proteins, and lipids, ultimately leading to cell death. Therefore, measuring ROS (Reactive Oxygen Species) production levels can be correlated with nematicide activity in *C. elegans*.

**Figure 13:**
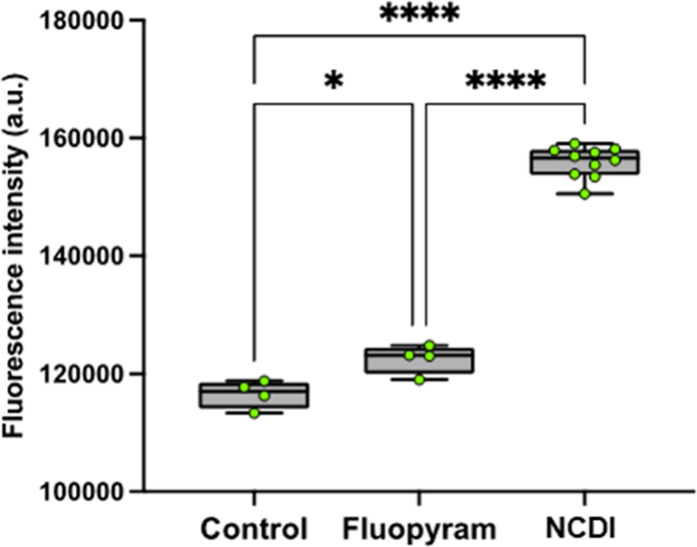
Oxidative burst measurement triggered by NCDI in *C. elegans* N2 by using the ROS indicator dihydrorhodamine-123 (DHR-123). Whisker plot showing the fluorescence intensity of *C. elegans* mitochondria, stained with DHR123 after treatment with 0.6 mM Fluopyram, 1 mM NCDI and 1.5% Acetone. Whiskers’ plot shows all measurements (green dots), medians (black line), and minimum and maximum (whiskers ends). Data sets passed Shapiro-Wilk test for normality (*P* > 0.05) and were compared using a parametric two-tailed Student′s *t* test with Welch’s correction.* *P* = 0.0122; **** *P* < 0.0001.

The effect of NCDI (1 mM) on ROS production in *C. elegans* was experimentally tested. NCDI exposure in *C. elegans* resulted in a substantial increase in ROS levels, 24 h after inoculation. Interestingly, this ROS upwelling was found to be significantly higher compared with the ROS levels induced by fluopyram (0.6 mM) (Figure 13). The heightened oxidative stress caused by NCDI suggests potential cellular damage.

### 3.7 *Caenorhabditis elegans* toxicity assay

The long-term impact of NCDI on *C. elegans* N2 lifecycle was evaluated by direct counting of the various stages of the lifecycle over a period of 5 days. The effect of NCDI (1 mM) was compared with the effect of both Fluopyram (0.6 mM) and Acetone (1.5%) acting as a positive and negative control, respectively.

The primary objective of this study was to assess various key stages in the *C.elegans* lifecycle, including egg numbers and the progression through different developmental phases (L1-L2, L3, L4, and adulthood), in response to NCDI exposure.

**Figure 14:**
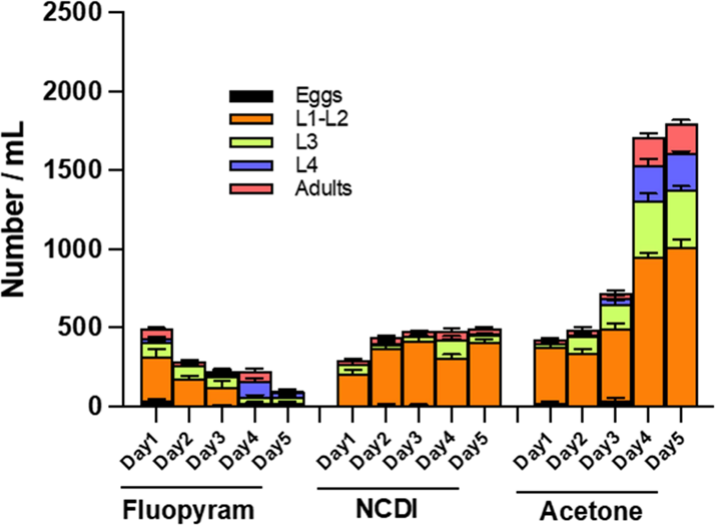
Effect of NCDI on C. elegans N2 lifecycle development. The impact of 0.6 mM fluopyram, 1 mM NCDI, and 1.5% acetone was assessed on the life cycle of C. elegans N2. This evaluation involved direct counting of various stages of the life cycle over a five-day duration.

Remarkably, NCDI (1 mM) exhibited a potent adverse effect on all stages of the *C. elegans* lifecycle, surpassing the impact of fluopyram (0.6 mM) within 24 h (Figure 14). This pronounced effect seemed to align with the observed induction of ROS. However, intriguingly, the severe impact of NCDI was not sustained over time. At the 2 d mark after inoculation, *C. elegans* demonstrated an increase in L1-L2 stages, which persisted throughout the duration of the experiment (Figure 14). This suggests that NCDI may have a nematostatic rather than a nematicidal activity, halting further development while not inducing death.

In contrast, the presence of acetone did not disrupt the normal progression of the life cycle as all stages exhibited an increase over the course of the experiment. Conversely, fluopyram displayed a gradual reduction in several stages of *C. elegans* lifecycle as time progressed (Figure 1).

To the best of our knowledge, this is the first study to successfully apply graph theory and computational chemistry methods to identify a new chemo-mathematical pattern that characterizes nematicide activity focusing on a new specific mode of action (CDA inhibition).

A novel candidate, NCDI, with promising nematicide activity has been identified, and future lead optimization studies and dose-dependent experimental *in vitro* assays are required to further understand the mechanistic profile of NCDI’s activity so as to consider its introduction into the agro market. Furthermore, the main strength of the present investigation lies in the computer-aided design strategy, which will enable the development of nematicides with a new MOA, from natural or semisynthetic origins and having different chemical scaffolds, having significant implications for the agri-food industry.

## Supporting Information Available

Supporting Information includes experimental results of the *Caenorhabditis elegans* toxicity assay for the fungal CDA, Topological descriptors value for DF_1_ (training set and LOO test set) and DF_2_ (training and test set), and a graphic representation of RMSD values for the Cα and heavy atoms of CDA *C. elegans* in complex with NCDI. (PDF)
