# Supplementary material for: Rational Design of a Potential New Nematicide Targeting Chitin Deacetylase
Source: J Agric Food Chem. 2024 Jan 24;72(5):2482–91. doi: 10.1021/acs.jafc.3c05258 (PMC10853968; doi:10.1021/acs.jafc.3c05258)
Supplement: Supplementary file 1 — jf3c05258_si_001.pdf [file jf3c05258_si_001.pdf]

RATIONAL DESIGN OF A POTENTIAL NEW NEMATICIDE TARGETING CHITIN DEACETYLASE

Maria Galvez-Llompart[a,b*], *Riccardo Zanni*[b], *David Vela-Corcía*[c], *Álvaro Polonio*[c], *Facundo Perez-Gimenez*[b], *Jesús Martínez-Cruz*[c], *Diego Romero*[c], *Dolores Fernández-Ortuño*[c], *Alejandro Pérez-García*[c†], *Jorge Galvez*[b†]

[a] Department of Preventive Medicine and Public Health, Food Science, Toxicology and Forensic Medicine, Faculty of Pharmacy, University of Valencia, 46100 Burjassot, Valencia, Spain

[b] Molecular Topology and Drug Design Unit. Department of Physical Chemistry, University of Valencia, 46100 Burjassot, Valencia, Spain

[c] Department of Microbiology, Faculty of Science, Instituto de Hortofruticultura Subtropical y Mediterránea La Mayora, IHSM-UMA-CSIC, University of Málaga, 29071, Málaga, Spain

†Co-last authors

*Corresponding author: maria.galvez@uv.es (M. Gálvez Llompart)

TABLE OF CONTENTS

Table S1. Experimental results of the *Caenorhabditis elegans* toxicity assay for the fungal CDA inhibitors selected during the previous work.[1] ……S3

Table S2. DF_1 values for each training set compound, along with each respective value of the equation's topological descriptors……S5

Table S3. LOO (leave one out) values for each training set compound, along with each respective value of the equation's topological descriptors………..S6

Table S4. DF_2 values for each training and test set compound, along with each respective value of the equation's topological descriptors………..S7

Figure S1. The RMSD values for the Cα atoms of CDA *C. elegans* in complex with NCDI were calculated over a 50 ns period of MD simulations (indicated by the blue lines). Additionally, the RMSD values were computed using the heavy atoms of the ligands, which were superimposed onto the Cα atoms of the protein through least-squares-fit, and are represented by the purple line………...……….S11

Table S1. Experimental results of the *Caenorhabditis elegans* toxicity assay for the fungal CDA inhibitors selected during the previous work.[1]

CODE	*Caenorhabditis elegans*	Larval stages					
		**L1**		**L2-L3**		**L4**	
	**COMPOUND**	**MEAN**	**EFFICACY* (%)**	**MEAN**	**EFFICACY (%)**	**MEAN**	**EFFICACY (%)**
	Water	2200.00[a]	-	3866.7	-	2333.33	-
	1.5% acetone	2533.33	0[b]	4933.33	0	2333.33	0
**#1 (VS#2-2)**	**(1-methyl-2,4-dioxo-1,4,6,7-tetrahydro[1,3]thiazole[2,3-f]purin-3(2H)-yl)acetic acid****	**4133.33**	**0**	**0**	**100**	**0**	**100**
#2	{3-[(1,3-dimethyl-2,4,6-trioxotetrahydro-5(2H)-pyrimidinylidene)methyl] phenoxy}acetic acid	1666.66	34.21	533.33	89.18	0	100
#3	5-[4-(2-hydroxyethoxy)-3-methoxybenzylidene]-1,3-dimethyl-2,4,6(1H,3H,5H)-pyrimidinetrione	2000	21.05	2400	51.35	0	100
#4	7-[2-hydroxy-3-(4-morpholinyl)propyl]-1,3-dimethyl-3,7-dihydro-1H-purine-2,6-dione	1800	28.94	800	83.78	66.67	97.14
#5	2-amino-7-methyl-5-oxo-4-[4-(trifluoromethoxy)phenyl]-4H,5H-pyrano[4,3-b]pyran-3-carbonitrile	2400	5.26	1466.67	70.27	0	100
#6	N-{4-[3-(2,6-dimethyl-4-morpholinyl)-2,5-dioxo-1-pyrrolidinyl]phenyl}acetamide	1533.33	39.47	1666,67	66.21	0	100
#7	2-(1,3-dimethyl-2,6-dioxo-1,2,3,6-tetrahydro-7H-purin-7-yl)-N-(2-methoxy-1-methylethyl)acetamide	1800	28.94	3800,00	22.97	1333.33	42.85
#8	N-cyclopropyl-2-(1,3-dimethyl-2,6-dioxo-1,2,3,6-tetrahydro-9H-purin-9-yl)acetamide	1866.67	26.31	3066.67	37.83	933.33	60
#9	3-benzyl-1,7-dimethyl-7,9-dihydro-1H-purine-2,6,8(3H)-trione	1600	36.84	2466.67	49.99	266.67	88.57
**#10 (VS#3-8)**	**5-imino-1-(2-methyl-5-nitrophenyl)-3-phenylhydantoin**	**5400**	**0**	**800**	**83.78**	**0**	**100**
#11	5-imino-1-(4-methyl-5-nitrophenyl)-3-phenylhydantoin	1933.33	23.68	4466.67	9.45	1133.33	51.42
#12	3-{2,5-dioxo-3-[(5Z)-4-oxo-5-(phenylmethylidene)-2-sulfanylidene-1,3-thiazolidin-3-yl]pyrrolidin-1-yl}propanoic acid	6133.33	0	866.67	82.43	466.67	79.99
**#13 (VS#3-2)**	**2-(10,12-dioxo-9-{[(1,3-thiazol-2-yl)carbamoyl]methyl}-7-thia-9,11-diazatricyclo[6.4.0.0^{2,6}]dodeca-1(8),2(6)-dien-11-yl)acetic acid**	**5066.67**	**0**	**666.67**	**86.48**	**0**	**100**
**#14 (VS#3-6)**	**2-{4-[(1,3-dimethyl-4,6-dioxo-2-sulfanylidene-1,3-diazinan-5-ylidene)methyl]phenoxy}acetic acid**	**4066,67**	**0**	**600**	**87.83**	**0**	**100**

**#15 (VS#3-1)**	**2-({[(5E)-4,6-dioxo-1-[4-(propan-2-yl)phenyl]-2-sulfanyl-1,4,5,6-tetrahydropyrimidin-5-ylidene]methyl}amino)-3-(1H-imidazol-4-yl)propanoic acid**	**4466.67**	**0**	**333.33**	**93.24**	**0**	**100**
#16	2-(2-methoxy-4-{[(5Z)-2,4,6-trioxo-1-(prop-2-en-1-yl)-1,3-diazinan-5-ylidene]methyl}phenoxy)acetic acid	-	-	-	-	-	-
#17	2-[(5E)-4-oxo-5-{[2-(prop-2-en-1-yloxy)phenyl]methylidene}-2-sulfanylidene-1,3-thiazolidin-3-yl]pentanedioic acid	3066.67	0	666.67	86.48	66.67	97.14
**#18 (VS#3-4)**	**2-{1-[(3,4-difluorophenyl)methyl]-3-oxopiperazin-2-yl}acetic acid**	**6066.67**	**0**	**400**	**91.89**	**0**	**100**
#19	2-{3-[(2-chloro-6-fluorophenyl)methyl]-2,4,5-trioxoimidazolidin-1-yl}acetic acid	3466.67	0	3933.33	20.27	1400	39.99
#20	{2-[(1,3-dimethyl-4,6-dioxo-2-thioxotetrahydro-5(2H)-pyrimidinylidene)methyl]phenoxy}acetic acid	2400	5.26	4266.67	13.51	1933.33	17.14

[a]The values represent the estimation of the number of individuals per ml.

[b]The values represent the percentage of efficacy of the compounds according to Abbott's formula.

* Efficacy (%) $= \frac{X-Y}{X} \times 100$, where X is the mean value of acetone (1.5%) and Y is the mean value of treatment with the corresponding compound (150 µM).

**Compounds in bold showed significant activity and have been selected as the initial training set for the development of the present QSAR strategy to identify new nematicides with activity against CDA.

Table S2. DF_1 values for each training set compound, along with each respective value of the equation's topological descriptors.

Compound	MATS5c	GATS3c	DF_1	Class.[1]	P.A.[2]
			**Active**		
**#01**	0.268	0.976	4.342	A	0.987
**#10**	0.071	1.104	-0.900	I	0.289
**#13**	0.500	0.995	8.295	A	1.000
**#14**	0.153	1.001	1.940	A	0.874
**#15**	0.017	0.832	1.686	A	0.844
**#18**	0.201	0.857	4.682	A	0.991
			**Inactive**		
**#02**	0.038	1.379	-5.085	I	0.006
**#03**	0.018	1.209	-3.232	I	0.038
**#04**	-0.100	1.071	-3.555	I	0.028
**#05**	-0.061	1.200	-4.544	I	0.011
**#06**	0.017	1.471	-6.676	I	0.001
**#07**	-0.159	1.147	-5.629	I	0.004
**#08**	0.167	1.290	-1.582	I	0.17
**#09**	-0.046	1.321	-5.851	I	0.003
**#11**	0.016	1.170	-2.759	I	0.06
**#12**	-0.222	1.033	-5.28	I	0.005
**#16**	-0.032	1.028	-1.767	I	0.146
**#17**	-0.010	1.040	-1.519	I	0.18
**#20**	0.084	1.117	-0.828	I	0.304
**#19**	0.032	0.865	1.531	A	0.822

[1] Classification according to LDA algorithm.

[2] Probability of activity, according to the discriminant function result.

Table S3. LOO (leave one out) values for each training set compound, along with each respective value of the equation's topological descriptors.

Compound	MATS5c	GATS3c	LOO DF_1	LOO Class.[1]	LOO P.A.[2]
			**Active**		
**#01**	0.268	0.976	4.342	A	0.984
**#10**	0.071	1.104	-0.900	I	0.151
**#13**	0.5	0.995	8.295	A	1
**#14**	0.153	1.001	1.940	A	0.859
**#15**	0.017	0.832	1.686	A	0.676
**#18**	0.201	0.857	4.682	A	0.988
			**Inactive**		
**#02**	0.038	1.379	-5.085	I	0.007
**#03**	0.018	1.209	-3.232	I	0.046
**#04**	-0.1	1.071	-3.555	I	0.035
**#05**	-0.061	1.2	-4.544	I	0.013
**#06**	0.017	1.471	-6.676	I	0.001
**#07**	-0.159	1.147	-5.629	I	0.004
**#08**	0.167	1.29	-1.582	I	0.254
**#09**	-0.046	1.321	-5.851	I	0.003
**#11**	0.016	1.17	-2.759	I	0.069
**#12**	-0.222	1.033	-5.280	I	0.005
**#16**	-0.032	1.028	-1.767	I	0.17
**#17**	-0.01	1.04	-1.519	I	0.201
**#19**	0.032	0.865	1.531	A	0.954
**#20**	0.084	1.117	-0.828	I	0.323

[1] Classification according to LDA LOO algorithm.

[2] Probability of activity, according to the LOO discriminant function result.

Table S4. DF_2 values for each training and test set compound, along with each respective value of the equation's topological descriptors.

Chemical or commercial name	CAS number	nSpiro	ATSC2i	SsssN	ETA_epsilon_3	DF_2	Class.[1]	P.A.[2]
**Active**								
**Furfural**	98-01-1	0.000	-4.819	0.000	0.433	-2.345	I	0.088
**Fenamiphos**	22224-92-6	0.000	14.033	0.000	0.433	-1.214	I	0.224
**Fluazaindolizine**	1254304-22-7	0.000	66.897	0.000	0.440	-0.347	I	0.382
**Ethoprofos**	13194-48-4	0.000	-7.997	0.000	0.427	-0.202	I	0.454
**Phorate**	298-02-2.	0.000	-3.707	0.000	0.427	0.056	A	0.516
**Iprodione**	36734-19-7	0.000	13.499	1.731	0.438	0.353	A	0.581
**Fosthiazate**	98886-44-3	0.000	0.489	1.415	0.433	0.563	A	0.637
**Aldoxycarb**	1646-88-4	0.000	14.019	0.000	0.427	0.960	A	0.718
**Temik**	116-06-3	0.000	17.275	0.000	0.426	1.499	A	0.812
**Aldicarb**	204-123-2	0.000	17.275	0.000	0.426	1.499	A	0.812
**Chloropicrin**	76-06-2	0.000	-2.195	0.000	0.422	1.973	A	0.879
**Fluopyram**	658066-35-4	0.000	106.001	0.000	0.437	3.045	A	0.944
**Avermectin B1**	65195-55-3	1.000	0.727	0.000	0.443	3.099	A	0.957
**Abamectin**	71751-41-2	1.000	0.727	0.000	0.443	3.099	A	0.957
**Oxamyl**	23135-22-0	0.000	14.656	1.350	0.427	3.468	A	0.969
**Metam Sodium**	137-42-8	0.000	3.065	0.000	0.418	3.756	A	0.977
**Azadirachtin**	189284-01-3.	1.000	47.426	0.000	0.447	4.382	A	0.986
**Benfuracarb**	82560-54-1	0.000	26.117	3.345	0.437	4.466	A	0.988
**Ethylene**	74-85-1	0.000	-4.403	0.000	0.414	4.514	A	0.989
**Dimethyl Disulfide**	624-92-0	0.000	-1.552	0.000	0.414	4.685	A	0.991
**Thiodicarb**	59669-26-0	0.000	20.841	2.201	0.429	4.695	A	0.991
**Spirotetramat**	203313-25-1	1.000	15.012	0.000	0.440	4.770	A	0.991
**Carbosulfan**	55285-14-8	0.000	25.456	3.800	0.437	5.166	A	0.994
**Imicyafos**	140163-89-9	0.000	17.818	3.616	0.433	5.631	A	0.996
**Carbon Disulfide**	75-15-0	0.000	0.090	0.000	0.409	6.648	A	0.999
**Dazomet**	533-74-4	0.000	31.677	4.296	0.433	7.706	A	1.000
**Methyl Iodide**	74-88-4	0.000	-3.137	0.000	0.400	9.716	A	1.000
**Inactive**								
**4-(Chloromethyl)-3-fluorobenzonitrile**	132305-92-1	0.000	-36.638	0.000	0.439	-6.207	I	0.002

**6-Oxoestriol (carboxymethyl)oxime**	37654-41-4	0.000	0.787	0.000	0.444	-5.836	I	0.003
**4-(4-Chlorobenzylidene)-2-(3-nitrophenyl)oxazol-5(4H)-one**	20345-11-3	0.000	-10.908	0.000	0.442	-5.654	I	0.004
**k-Strophantoside**	33279-57-1	0.000	7.075	0.000	0.444	-5.594	I	0.004
**Benzene,1-[bis(phenylthio)methyl]-4-nitro**	23837-16-3	0.000	-9.150	0.000	0.441	-5.419	I	0.005
**N,N'-bis(cyclohexyloxycarbonyl)-4-methyl-1,3-phenylenediamine**	30714-89-7	0.000	-14.522	0.000	0.440	-5.412	I	0.005
**1-methoxythianthrene**	109014-97-3	0.000	17.350	0.000	0.445	-5.364	I	0.005
**Erythrosin B**	16423-68-0	0.000	-0.039	0.000	0.443	-5.357	I	0.005
**2-Chloro-6-(3-hydroxypropylamino)-1-phenalenone**	113722-81-9	0.000	2.045	0.000	0.443	-5.350	I	0.005
**Bisphenol AP**	1571-75-1	0.000	-2.763	0.000	0.442	-5.308	I	0.005
**4-Hydroxy-2-phenyl-6-quinolinesulfonyl fluoride**	31241-71-1	0.000	1.404	0.000	0.442	-5.215	I	0.005
**N-(4-methylphenyl)-2,3-dihydrothiophen-3-amine 1,1-dioxide**	39565-71-4	0.000	-14.821	0.000	0.440	-5.170	I	0.006
**Quinazoline-4-thiol**	3337-86-8	0.000	1.252	0.000	0.442	-5.067	I	0.006
**2-(2-Thiazolylazo)-p-cresol**	1823-44-5	0.000	-12.873	0.000	0.440	-5.053	I	0.007
**2-Amino-4-methylbenzothiazole**	1477-42-5	0.000	1.759	0.000	0.442	-5.036	I	0.006
**Corticosterone 21-Acetate**	1173-26-8	0.000	13.962	0.000	0.444	-4.898	I	0.007
**allyl 1-benzotriazolyl carbonate**	102423-16-5	0.000	-10.814	0.000	0.439	-4.784	I	0.008
**N-(2,2-dichloro-1-(4-chloro-benzenesulfonyl)-ethyl)-benzamide**	136800-79-8	0.000	-12.323	0.000	0.437	-4.223	I	0.015
**4-chloro-4'-cyanobenzenesulfonanilide**	134899-80-2	0.000	-6.285	0.000	0.438	-4.172	I	0.015
**8-Amino-2-naphthalenesulfonic acid**	119-28-8	0.000	2.255	0.000	0.440	-4.145	I	0.016
**5-Amino-2-naphthalenesulfonic acid**	119-79-9	0.000	2.255	0.000	0.440	-4.145	I	0.016
**1,3-Bis(2-methoxyphenyl)thiourea**	1226-64-8	0.000	-6.959	0.000	0.438	-4.123	I	0.016
**6-(3-chloro-2-hydroxy-propylamino)-2-phenyl-benzo(de)isoquinoline-1,3-dione**	37415-77-3	0.000	-4.405	1.210	0.444	-3.933	I	0.019
**Fumonisin B_1**	116355-83-0	0.000	-38.882	0.000	0.432	-3.759	I	0.025
**ethyl 4-(4-methoxyphenyl)-6-methyl-2-thioxo-1,2,3,4-tetrahydro-5-pyrimidinecarboxylate**	113697-57-7	0.000	-1.793	0.000	0.438	-3.732	I	0.024

**6,8-diiodo-4-quinazolone**	100540-61-2	0.000	16.510	0.000	0.441	-3.651	I	0.025
**carbobenzyloxyvalylalanine benzyl ester**	118234-89-2	0.000	-2.422	0.000	0.436	-3.288	I	0.036
**Carbobenzyloxy-L-isoleucylglycine benzyl ester**	118402-78-1	0.000	-0.234	0.000	0.436	-3.157	I	0.041
**1-(2-amino-phenyl)-pyrrolidine-2,5-dione**	1012-81-3	0.000	-13.068	1.162	0.440	-3.105	I	0.044
**5-benzamidovaleric acid**	15647-47-9	0.000	-9.381	0.000	0.433	-2.619	I	0.069
**Cyclohex-3-enone**	4096-34-8	0.000	-9.214	0.000	0.433	-2.608	I	0.070
**tyrphostin 47**	118409-60-2	0.000	-8.197	0.000	0.433	-2.547	I	0.074
**4'-ethoxycarbonylpropionanilide**	132371-06-3	0.000	-7.641	0.000	0.433	-2.514	I	0.076
**4-Chlorophenyl 2-bromoethyl ether**	2033-76-3	0.000	-6.844	0.000	0.433	-2.466	I	0.079
**2-Ethylfuran**	3208-16-0	0.000	-5.516	0.000	0.433	-2.387	I	0.085
**2-Thiophenecarbonitrile**	1003-31-2	0.000	-2.629	0.000	0.433	-2.213	I	0.099
**3,4-dichloro-5-methyl-2(5h)-furanone**	19026-11-0	0.000	-1.852	0.000	0.433	-2.167	I	0.103
**3,4-Dibromo-3-methyltetrahydrothiophene 1,1-dioxide**	17536-53-7	0.000	-1.701	0.000	0.433	-2.158	I	0.104
**3-Methyl-2-cyclopenten-1-one**	2758-18-1	0.000	-1.563	0.000	0.433	-2.149	I	0.105
**4-Chlorophenyl 2-chloro-1,1,2-trifluoroethyl sulfone**	26574-59-4	0.000	-1.405	0.000	0.433	-2.140	I	0.106
**2-[(tert-butylamino)carbonyl]benzoic acid**	20320-35-8	0.000	-0.781	0.000	0.433	-2.103	I	0.109
**3-dodecylsulfanyl-propionamide**	21790-36-3	0.000	-26.319	0.000	0.429	-1.926	I	0.134
**n-(2-(3,4-dimethoxy-phenyl)-ethyl)-propionamide**	20944-13-2	0.000	24.395	0.000	0.437	-1.803	I	0.136
**2-(Dodecylsulfinyl)ethanol**	20413-40-5	0.000	-25.846	0.000	0.428	-1.801	I	0.149
**N-(Benzyloxycarbonyl)-2-aminoacetonitrile**	3589-41-1	0.000	4.265	0.000	0.433	-1.800	I	0.141
**ethyl 4-carbamimidoylbenzoate**	15676-12-7	0.000	4.637	0.000	0.433	-1.777	I	0.144
**linolenelaidic acid methyl ester**	14202-25-6	0.000	-19.300	0.000	0.429	-1.742	I	0.154
**5-formyl-2,4,6-trimethyl-nicotinic acid ethyl ester**	28569-08-6	0.000	5.931	0.000	0.433	-1.700	I	0.153
**2-(dodecylthio)ethanol**	1462-55-1	0.000	-24.431	0.000	0.428	-1.608	I	0.174
**Trimethylolpropane Tris(thioglycolate)**	10193-96-1	0.000	-7.175	0.000	0.429	-1.014	I	0.269

**4-(4-methoxy-phenyl)-4-trifluoromethyl-(1,5,3)oxathiazepan-2-one**	154541-47-6	0.000	64.612	0.000	0.438	0.171	A	0.510
**4-Ethoxyfluorophosphinyloxy tempo**	37566-53-3	0.000	7.806	1.254	0.433	0.707	A	0.666
**N-((3-Ethyl-4-oxo-2-thioxo-5-thiazolidinylidene)methyl)**	3747-06-6	0.000	-1.364	2.983	0.438	1.672	A	0.842
**Chloral hydrate**	302-17-0	0.000	-4.348	0.000	0.422	1.843	A	0.865
**L-732138**	148451-96-1	0.000	114.688	0.000	0.439	2.811	A	0.929
**Azadirachtin**	11141-17-6	1.000	47.426	0.000	0.447	4.382	A	0.986
**Test active**								
**Methyl Bromide**	1111-88-2	0.000	-0.335	0.000	0.400	9.884	A	1.000
**Metam sodium**	137-42-8	0.000	18.83	0.000	0.418	4.702	A	0.990
**DBCP**	67708-83-2	0.000	-3.282	0.000	0.420	2.531	A	0.927
**Milbemectin**	1799297-76-9	1.000	-5.398	0.000	0.443	2.420	A	0.92
**Terbufos**	13071-79-9	0.000	12.455	0.000	0.428	0.727	A	0.669
**Cadusafos**	95465-99-9	0.000	3.194	0.000	0.428	0.172	A	0.542
**Fluensulfone**	318290-98-1	0.000	-0.934	0.000	0.433	-2.112	I	0.108
**Carbofuran**	1563-66-2	0.000	16.405	0.000	0.439	-3.151	I	0.040
**Test inactive**								
**1-(methylthio)isoquinolin-3(4h)-one**	36068-76-5	0.000	-6.115	0.000	0.441	-5.008	I	0.007
**7-benzyloxy-9-(4-benzyloxy-3-meo-phenyl)-6-methoxy-3h-naphtho(2,3-c)furan-1-one**	38153-36-5	0.000	27.467	0.000	0.445	-4.757	I	0.008
**2,3-Dimethyl-5-nitroindole**	21296-94-6	0.000	-3.845	0.000	0.440	-4.678	I	0.009
**hexadecyl-(2-nitro-phenyl)-sulfide)**	113251-93-7	0.000	-28.529	0.000	0.433	-3.767	I	0.024
**6-(2-benzoylamino-3-(4-methoxy-phenyl)-acryloylamino)-hexanoic acid**	150890-94-1	0.000	-10.132	0.000	0.436	-3.751	I	0.023
**4-(4-acetamidophenyl)-1,3-dithiole-2-thione**	13575-06-9	0.000	6.618	0.000	0.439	-3.739	I	0.023
**Chromoionophore V**	132097-01-9	0.000	-6.470	2.266	0.447	-3.178	I	0.041
**Carbofuran**	1563-66-2	0.000	16.405	0.000	0.439	-3.151	I	0.040
**2-(benzylsulfanyl)acetonitrile**	17377-30-9	0.000	-11.354	0.000	0.433	-2.737	I	0.062
**N-(4-Cyanomethyl-2-nitro-phenyl)-acetamide**	123270-23-5	0.000	-10.592	0.000	0.433	-2.691	I	0.065

**carbobenzyloxy-l-methionyl-l-valinamide**	114702-63-5	0.000	-10.017	0.000	0.433	-2.657	I	0.067
**Phenylurethane**	101-99-5	0.000	-2.539	0.000	0.433	-2.208	I	0.100
**2-[N,N-Bis(trifluoromethanesulfonyl)amino]-5-chloropyridine**	145100-51-2	0.000	54.664	-1.837	0.433	-2.138	I	0.096
**4-(Fluorosulfonyl)benzoyl chloride**	402-55-1	0.000	10.416	0.000	0.433	-1.431	I	0.190
**N,S-Diacetyl-L-cysteine methyl ester**	19547-88-7	0.000	-4.660	0.000	0.427	-0.161	I	0.463
**methyl (2-chloro-1-methylethylidene)hydrazinecarboxylate**	103517-77-7	0.000	-4.188	0.000	0.425	0.683	A	0.667
**Hycanthone**	3105-97-3	0.000	23.079	2.341	0.441	0.917	A	0.705
**Nigericin**	28380-24-7	1.000	-6.445	0.000	0.443	2.622	A	0.934

[1] Classification according to LDA algorithm.

[2] Probability of activity, according to the discriminant function result.

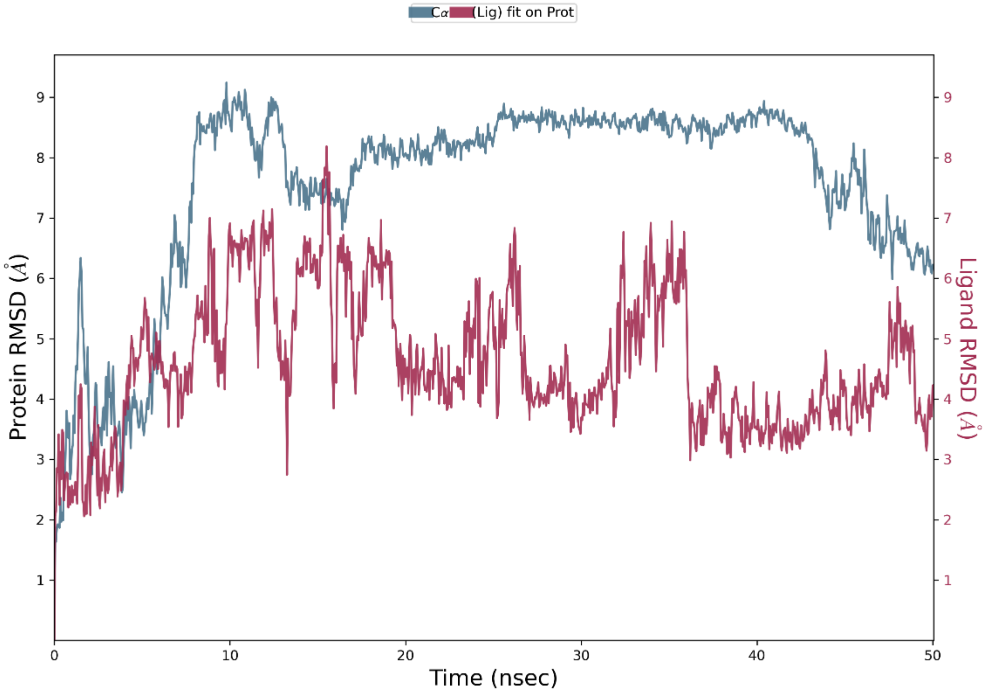

Figure S1. The RMSD values for the Cα atoms of CDA *C. elegans* in complex with NCDI were calculated over a 50 ns period of MD simulations (indicated by the blue lines). Additionally, the RMSD values were computed using the heavy atoms of the ligands, which were superimposed onto the Cα atoms of the protein through least-squares-fit, and are represented by the purple line.

REFERENCE

1. Zanni, R.; Martínez-Cruz, J.; Gálvez-Llompart, M.; Fernández-Ortuño, D.; Romero, D.; García-Domènech, R.; Pérez-García, A.; Gálvez, J. Rational Design of Chitin Deacetylase Inhibitors for Sustainable Agricultural Use Based on Molecular Topology. *J. Agric. Food Chem.* **2022,** *70*, 13118-13131.
